# Associations of five dietary indices with metabolic dysfunction-associated steatotic liver disease and liver fibrosis among the United States population

**DOI:** 10.3389/fnut.2024.1446694

**Published:** 2024-08-16

**Authors:** Min Xu, Yamei Zhan, Guohui Gao, Li Zhu, Tong Wu, Guijie Xin

**Affiliations:** ^1^Department of Hepatology, Center of Infectious Diseases and Pathogen Biology, The First Hospital of Jilin University, Changchun, China; ^2^Department of Nephrology, The First Hospital of Jilin University, Changchun, China

**Keywords:** MASLD, NAFLD, NHANES, dietary index, liver fibrosis

## Abstract

**Background and aims:**

The role of dietary factors in metabolic dysfunction-associated steatotic liver disease (MASLD)—which represents a new definition of liver steatosis and metabolic dysfunction— remains unclear. This study aimed to explore the relationships between dietary indices and MASLD.

**Methods:**

We analyzed data from the United States National Health and Nutrition Examination Survey (NHANES) 2017–2020 cycle, including 4,690 participants with complete vibration-controlled transient elastography (VCTE) data. Multivariate logistic regression models adjusted for covariates were used to assess the association between dietary indices, MASLD, and MASLD-associated liver fibrosis (MASLD-LF). Restricted cubic spline (RCS) models and subgroup analyses were also performed.

**Results:**

The Alternative Healthy Eating Index (AHEI), Healthy Eating Index-2020 (HEI-2020), Dietary Approaches to Stop Hypertension Index (DASHI), and Mediterranean Diet Index (MEDI) were found to be negatively associated with MASLD risk, while the Dietary Inflammatory Index (DII) had a positive association. The highest quartile of MEDI was linked to a 44% reduction in MASLD risk [Q1 vs. Q4 odds ratio (OR): 0.56; 95% confidence interval (CI): 0.34–0.94, P for trend: 0.012]. DASHI was uniquely associated with a reduced risk of MASLD-LF (continuous OR: 0.79; 95% CI: 0.64–0.97; *p* for trend: 0.003). Our RCS curves indicated a nonlinear association with DASHI-MASLD (*p*-overall: 0.0001, p-nonlinear: 0.0066). Subgroup analyses showed robust associations among the non-Hispanic White and highly educated populations.

**Conclusion:**

Specific dietary patterns were associated with reduced risks of MASLD and MASLD-LF. The DASHI, in particular, showed a significant protective effect against MASLD-LF. These findings suggest potential dietary interventions for managing MASLD and MASLD-LF, although large-scale randomized controlled trials are warranted to validate these findings.

## 1 Introduction

Over recent decades, non-alcoholic fatty liver disease (NAFLD) has emerged as the leading cause of chronic liver disease worldwide, affecting 30% of adults across the planet (1). Owing to certain limitations in the definition of NAFLD, such as its exclusive diagnostic criteria and the stigmatization of the word “fatty” (2), a recent expert panel adopted new terms and definitions for liver disease—ultimately replacing the term NAFLD with metabolic dysfunction-associated steatotic liver disease (MASLD) (3).

The disease spectrum of MASLD includes simple steatosis, steatohepatitis, fibrosis, and cirrhosis. Its high prevalence makes MASLD a leading cause of end-stage liver disease, liver transplantation, and liver-related mortality (4). However, no specific pharmaceutical medications are currently available to treat MASLD. Lifestyle modifications therefore remain an important approach for improving MASLD. Diet, weight loss, and physical activity are considered the cornerstones of MASLD treatment (5), with dietary factors considered to play a key role in its pathogenesis and a high-quality diet being considered a potential prophylactic strategy (6). Nevertheless, previous guidelines regarding the dietary recommendations for patients with MASLD have been relatively vague or nonspecific (7, 8), unlike those for patients with type 2 diabetes.

Dietary pattern indices consider the contributions of various aspects of diet, including nutrients and food quality. As a result, they can more closely simulate real-world scenarios of nutrient and food combinations, facilitating the translation of clinical findings into dietary recommendations (9). Recently, several dietary indices have been developed to explore the associations between dietary quality and health outcomes (10). These indices include the Alternative Healthy Eating Index (AHEI), Healthy Eating Index-2020 (HEI-2020), Dietary Approaches to Stop Hypertension Index (DASHI), Dietary Inflammatory Index (DII), and Mediterranean diet Index (MEDI) (11–16). Several studies have predicted NAFLD risk according to different dietary indices (10, 17); however, the applicability of these findings to MASLD remains uncertain.

This study aimed to explore the effects of various dietary patterns on MASLD and MASLD-associated liver fibrosis (MASLD-LF) adopting the latest definition of MASLD and dietary pattern indices.

## 2 Methods

### 2.1 Study population

The National Health and Nutrition Examination Survey (NHANES) is a population-based cross-sectional survey designed to collect health and nutritional information about the U.S. population. It includes demographic data, participant examinations, laboratory data, and information concerning health and nutritional statuses. Written informed consent was obtained from all of the participants before any data were collected, and all of the study protocols were approved by the National Center for Health Statistics’ ethical review board.

We extracted data from the NHANES 2017–2020 cycle, because it contained data regarding vibration-controlled transient elastography (VCTE). VCTE is a commonly used non-invasive examination that measures the controlled attenuation parameter (CAP) and liver stiffness measurement (LSM) to assess liver steatosis and fibrosis (18).

**Figure 1:**
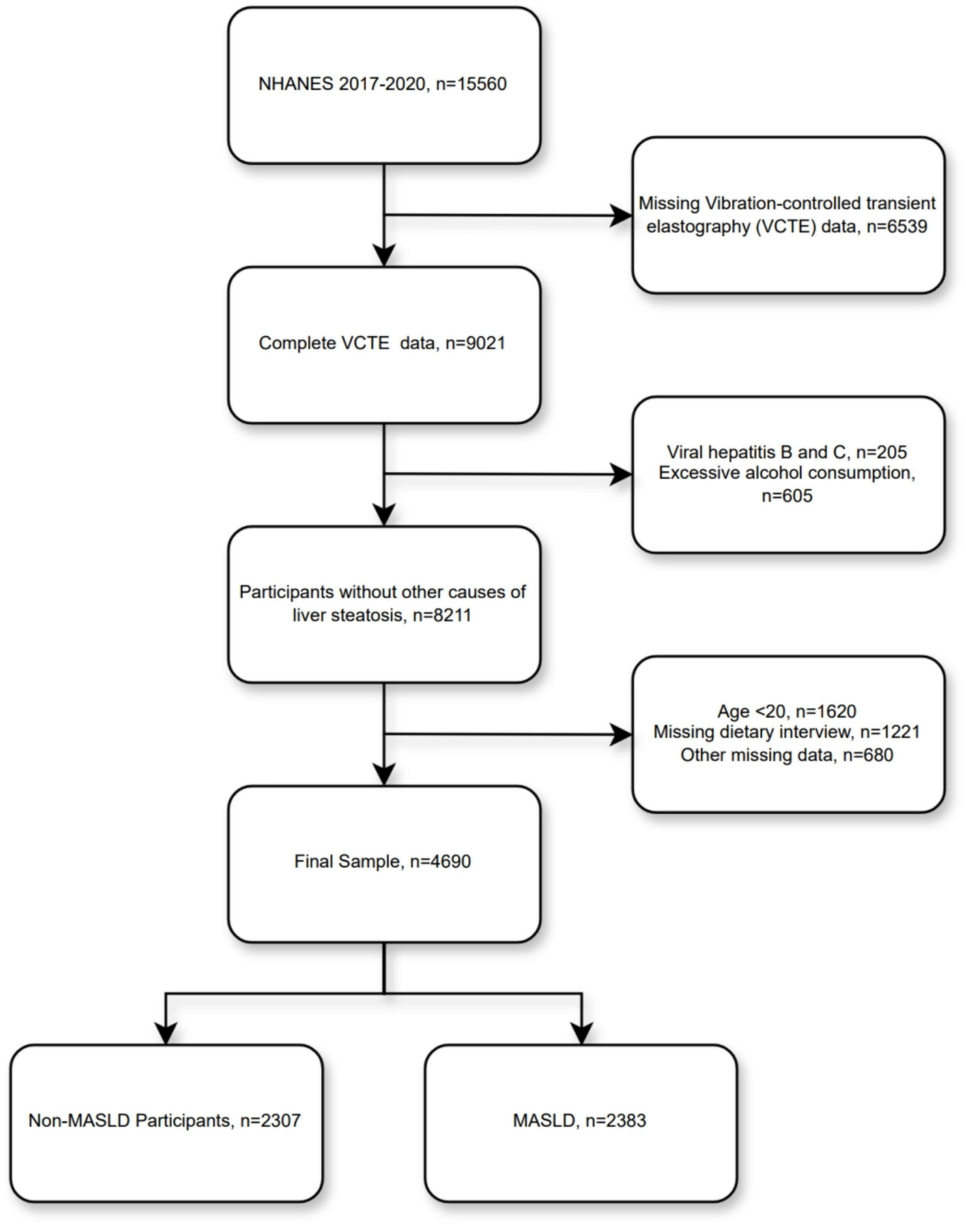
Flowchart of the study population selection.

A total of 15,560 individuals participated in this cycle, of whom 9,021 had complete VCTE results. Subsequently, we excluded other causes of steatotic liver disease (205 participants with viral hepatitis B and C, and 605 with excessive alcohol consumption), 1,620 underage participants, 1,221 participants with missing dietary interview data, and 680 participants with other missing data—resulting in a final total of 4,690 participants included in our analysis (Figure 1).

### 2.2 Metabolic dysfunction-associated steatotic liver disease

VCTE was performed, and CAP and LSM values were measured to assess hepatic steatosis and fibrosis. For each participant, CAP ≥264 dB/m was defined as liver steatosis (19), and LSM ≥8.0 kPa was defined as fibrosis (20). MASLD was defined as patients with liver steatosis who excluded excessive alcohol consumption [≥30/20 g/day (male/female)] and viral hepatitis (hepatitis B Surface Antigen/hepatitis C antigen/hepatitis C RNA positive) and fulfilled one of the following cardiometabolic risk factors: (1) body mass index (BMI) ≥25 kg/m^2^ (23 kg/m^2^ for Asia) or waist circumference (WC) ≥94/80 cm (male/female); (2) fasting blood glucose ≥100 mg/dL or glycosylated hemoglobin ≥5.7%), or type 2 diabetes, or undergoing treatment for type 2 diabetes; (3) blood pressure ≥ 130/85 mmHg or undergoing antihypertensive treatment; (4) plasma triglycerides ≥150 mg/dL or undergoing lipid-lowering treatments; (5) plasma high-density lipoprotein cholesterol (HDL-C) <40 mg/dL for men or < 50 mg/dL for women, or receiving lipid-lowering treatments (2).

### 2.3 Dietary indices

All dietary indices in this study were calculated based on information from the two-day, 24-h dietary interviews conducted by the NHANES (Supplementary Table 1). The HEI-2020, the most recent version of the HEI, measures the quality of an individual’s dietary patterns, independent of quantity, and has been evaluated for consistency with the Dietary Guidelines for Americans (DGA) (15). The HEI-2020 was calculated based on 13 dietary components—including nine adequacy components and four moderation components. For the moderating components, higher scores were associated with lower levels of consumption. The total score ranged between 0 and 100, with higher scores indicating higher dietary quality. Similarly, the AHEI consists of 11 dietary components, with each component accounting for 0–10 points and a total score ranging between 0 and 110. This score assesses diet quality and risk of chronic diseases based on an individual’s food and nutrient intake (14).

DII is a score that quantifies the association between an individual’s dietary intake and six inflammatory factors, namely interleukin-1beta (IL-1β), IL-4, IL-6, IL-10, tumor necrosis factor-alpha (TNF-α), and C-reactive protein (16). The DII was designed to measure the inflammatory potential of a given diet, with higher scores indicating a more pro-inflammatory diet and lower scores indicating a more anti-inflammatory one (21).

The DASHI is a dietary index based on nine nutrients (protein, fiber, magnesium, calcium, potassium, total fat, saturated fat, cholesterol, and sodium) (22), and is calculated based on 1-day nutrient consumption. All nutrients were divided by total energy/2000 kcal, to adjust for energy intake (11). The total DASHI score ranges between 0 and 9, with higher scores suggesting a dietary pattern that tends to prevent hypertension.

The MED differs from the conventional Western diet, which is rich in red meat, refined grains, and sugar-sweetened beverages (SSB). It emphasizes the intake of fruits, vegetables, whole grains, nuts, peanuts, olive oil, and seafood (6). The MEDI is calculated based on the serving sizes of these food equivalents per day. The additional inclusion of levels of intake of SSB, sweets, discretionary fats, and red meat—all of which are not recommended in the MED—improves the reliability of the score (12).

### 2.4 Covariates

Based on previous studies, several MASLD-related covariates were included: sex, age, ethnicity (Mexican-American, Other Hispanic, Non-Hispanic White, Non-Hispanic Black, Non-Hispanic Asian, Other/Multiracial), education level (<High school, ≥High school), BMI grade (obesity: ≥30 kg/m^2^, overweight: 25–30 kg/m^2^, and normal weight: <25 kg/m^2^), smoking status (never smoker, former smoker, or current smoker), hypertension, diabetes, total cholesterol (TC), total triglycerides (TG), and HDL-C (23). Diabetes was defined as fasting blood glucose of ≥126 mg/dL or glycated hemoglobin ≥6.5%, self-reported diabetes mellitus, or receiving insulin treatment (24). Participants with systolic blood pressure measurements of ≥130 mmHg or diastolic blood pressure measurements of ≥80 mmHg, self-reported hypertension, or those taking antihypertensive medication were defined as having hypertension (23).

### 2.5 Statistical analysis

Continuous variables are expressed as means (standard errors [SEs]), and categorical ones are expressed as numbers and percentages. Comparisons between groups were performed using Student’s t-tests and Chi-squared tests with complex survey samples. Multivariate logistic regression analyses were performed to explore the effects of different dietary indices on MASLD and MASLD-LF. All dietary indices were divided into quartiles for trend testing. Three models were used: Model 1 (unadjusted); Model 2 (adjusted for age, sex, ethnicity, and educational level); and Model 3 (adjusted for the variables in Model 2, as well as BMI grade, smoking status, hypertension, diabetes, TG, TC, and HDL-C). Nonlinear associations between the five dietary indices and MASLD and MASLD-LF scores were explored using a four-knot-restricted cubic spline curve (RCS). Subgroup analyses and interaction tests were performed for different populations based on categorical covariates (sex, age [<50, ≥50], ethnicity, educational level, BMI grade, smoking status, hypertension, and diabetes). According to the NHANES recommendations, unequal probability of selection and non-response were accounted for using “WTMECPRP” weights. Statistical analysis and data visualization were performed using R 4.2.3 software, and statistical significance was set at *p* < 0.05.

## 3 Results

### 3.1 Baseline population characteristics

A total of 4,690 participants were involved in this study. They had a mean age of 48.63 ± 0.62 years, 47.47% were male, 2,383 had MASLD (weighted prevalence, 49.30%), and 375 had MASLD-LF (weighted prevalence, 15.81%). As for dietary scores, AHEI [39.26 (0.45)], HEI [50.19 (0.41)], DASHI [3.29 (0.03)], and MEDI [3.60 (0.04)] levels were generally low, none of them reaching the median. The mean DII [1.22 (0.66)] exceeded the value of 0 suggesting that the diet was pro-inflammatory. At baseline, there were significant differences in most variables between the non-MASLD and MASLD groups. Compared to the population without MASLD population, the one with MASLD had a higher proportion of males (51.73% vs. 43.33%), an older mean age (51.93 ± 0.74 vs. 45.43 ± 0.73), a higher proportion of Mexican-Americans (10.94% vs. 5.66%), and a lower proportion of individuals with more than a high school educational level (89.26% vs. 91.76%). The population with MASLD had a higher prevalence of diabetes and hypertension; higher BMI, WC, alanine aminotransferase (ALT), aspartate aminotransferase (AST), TC, TG, CAP, LSM, AHEI, HEI-2020, DASHI, and MEDI levels; and lower HDL and DII levels (all *p* < 0.05). There were no significant differences in terms of age, sex, ethnicity, educational level, smoking status, TC, TG, or DII between the participants with non-fibrotic MASLD and those with MASLD-LF. However, significant differences were observed in terms of the other variables. Table 1 summarizes the clinical characteristics of the participants.

**Table 1:** Characteristics of the study population.

Characteristics	Overall (*n* = 4,690)	Non-MASLD (*n* = 2,307)	MASLD (*n* = 2,383)	*p*-value*	Non-Fibrosis MASLD (*n* = 2008)	MASLD-LF (*n* = 375)	*p*-value*
Age (years)	48.63 (0.62)	45.43 (0.73)	51.93 (0.74)	<0.001	51.68 (0.75)	53.24 (1.15)	0.147
Sex				<0.001			0.544
Female	2,495 (52.53%)	1,319 (56.67%)	1,176 (48.27%)		1,004 (48.70%)	172 (45.98%)	
Male	2,195 (47.47%)	988 (43.33%)	1,207 (51.73%)		1,004 (51.30%)	203 (54.02%)	
Race				<0.001			0.315
Non-Hispanic White	1,656 (63.75%)	767 (63.84%)	889 (63.67%)		738 (62.88%)	151 (67.86%)	
Non-Hispanic Black	1,245 (11.35%)	730 (13.65%)	515 (9.00%)		436 (9.23%)	79 (7.75%)	
Mexican American	564 (8.26%)	185 (5.66%)	379 (10.94%)		317 (11.07%)	62 (10.27%)	
Non-Hispanic Asian	520 (5.31%)	280 (5.65%)	240 (4.97%)		214 (7.28%)	34 (6.17%)	
Other Hispanic	483 (7.40%)	235 (7.68%)	248 (7.11%)		212 (5.29%)	28 (3.28%)	
Other/multiracial	222 (3.92%)	110 (3.52%)	112 (4.32%)		91 (4.26%)	21 (4.66%)	
Education				0.004			0.766
<High School	761 (9.47%)	328 (8.24%)	433 (10.74%)		370 (10.84%)	63 (10.23%)	
High School or above	3,929 (90.53%)	1,979 (91.76%)	1,950 (89.26%)		1,638 (89.16%)	312 (89.77%)	
Hypertension	2,700 (51.80%)	1,082 (38.00%)	1,618 (65.98%)	<0.001	1,314 (63.07%)	304 (81.45%)	<0.001
Diabetes	950 (15.73%)	229 (5.90%)	721 (25.84%)	<0.001	527 (21.58%)	194 (48.52%)	<0.001
Smoking status				0.002			0.108
Current smokers	729 (13.41%)	406 (14.97%)	323 (11.80%)		277 (12.01%)	46 (10.69%)	
Former smokers	1,105 (25.86%)	443 (21.90%)	662 (29.93%)		538 (28.70%)	124 (36.45%)	
Nonsmoker	2,856 (60.73%)	1,458 (63.13%)	1,398 (58.27%)		1,193 (59.29%)	205 (52.86%)	
BMI grade				<0.001			<0.001
Normal (<25)	1,115 (25.05%)	949 (43.74%)	166 (5.84%)		157 (6.70%)	9 (1.23%)	
Obesity (≥30)	2,087 (43.59%)	568 (21.85%)	1,519 (65.95%)		1,213 (62.21%)	306 (85.85%)	
Overweight (25–30)	1,488 (31.35%)	790 (34.40%)	698 (28.22%)		638 (31.09%)	60 (12.92%)	
BMI (kg/m2)	29.89 (0.22)	26.46 (0.20)	33.41 (0.27)	<0.001	32.48 (0.30)	38.39 (0.50)	<0.001
WC (cm)	100.92 (0.57)	91.91 (0.52)	110.20 (0.64)	<0.001	107.97 (0.65)	122.04 (1.17)	<0.001
ALT (U/L)	22.16 (0.27)	18.62 (0.38)	25.80 (0.51)	<0.001	24.52 (0.53)	32.62 (1.42)	<0.001
AST (U/L)	21.14 (0.16)	20.24 (0.25)	22.07 (0.29)	<0.001	21.18 (0.28)	26.82 (1.13)	<0.001
TC (mg/dL)	187.54 (1.41)	185.19 (1.58)	189.96 (1.77)	0.014	190.74 (1.85)	185.77 (2.62)	0.077
TG (mg/dL)	139.77 (2.65)	108.65 (1.72)	171.76 (3.66)	<0.001	167.49 (3.74)	194.48 (15.02)	0.103
HDL (mg/dL)	52.58 (0.45)	57.15 (0.56)	47.89 (0.47)	<0.001	48.37 (0.48)	45.34 (0.87)	0.002
CAP (dB/m)	264.66 (1.72)	213.81 (1.17)	316.96 (1.36)	<0.001	312.78 (1.23)	339.21 (2.82)	<0.001
LSM (kPa)	5.79 (0.12)	4.91 (0.08)	6.70 (0.21)	<0.001	5.17 (0.05)	14.83 (0.99)	<0.001
HEI-2020	50.19 (0.41)	51.35 (0.54)	48.99 (0.38)	<0.001	49.51 (0.42)	46.21 (0.94)	0.005
AHEI	39.26 (0.45)	40.50 (0.58)	37.98 (0.41)	<0.001	38.39 (0.43)	35.79 (0.87)	0.011
DASHI	3.29 (0.03)	3.40 (0.04)	3.17 (0.04)	<0.001	3.23 (0.04)	2.88 (0.08)	<0.001
DII	1.22 (0.06)	1.13 (0.08)	1.31 (0.06)	0.011	1.30 (0.07)	1.39 (0.14)	0.582
MEDI	3.60 (0.04)	3.70 (0.05)	3.50 (0.04)	<0.001	3.53 (0.04)	3.35 (0.08)	0.018

Continuous variables were expressed as mean (SE), and categorical variables were expressed as numbers (percentage). *Comparisons between groups were performed using *t*-tests and chi-square tests with complex survey samples. MASLD: metabolic dysfunction-associated steatotic liver disease, MASLD-LF, MASLD-associated liver fibrosis; BMI, body mass index; WC, waist circumstance; MASLD, metabolic dysfunction-associated steatotic liver disease; ALT, alanine aminotransferase; AST, aspartate aminotransferase; TC, total cholesterol; TG, total triglycerides; HDL-C, high-density lipoprotein cholesterol; HEI-2020, Healthy Eating Index-2020; AHEI, Alternate Healthy Eating Index; DASHI, Dietary Approaches to Stop Hypertension index in serving sizes adapted from the DASH trial; DII, Dietary Inflammatory Index; MED, Mediterranean diet index in serving sizes from the PREDIMED trial.

### 3.2 Association of dietary indices with MASLD

**Figure 2:**
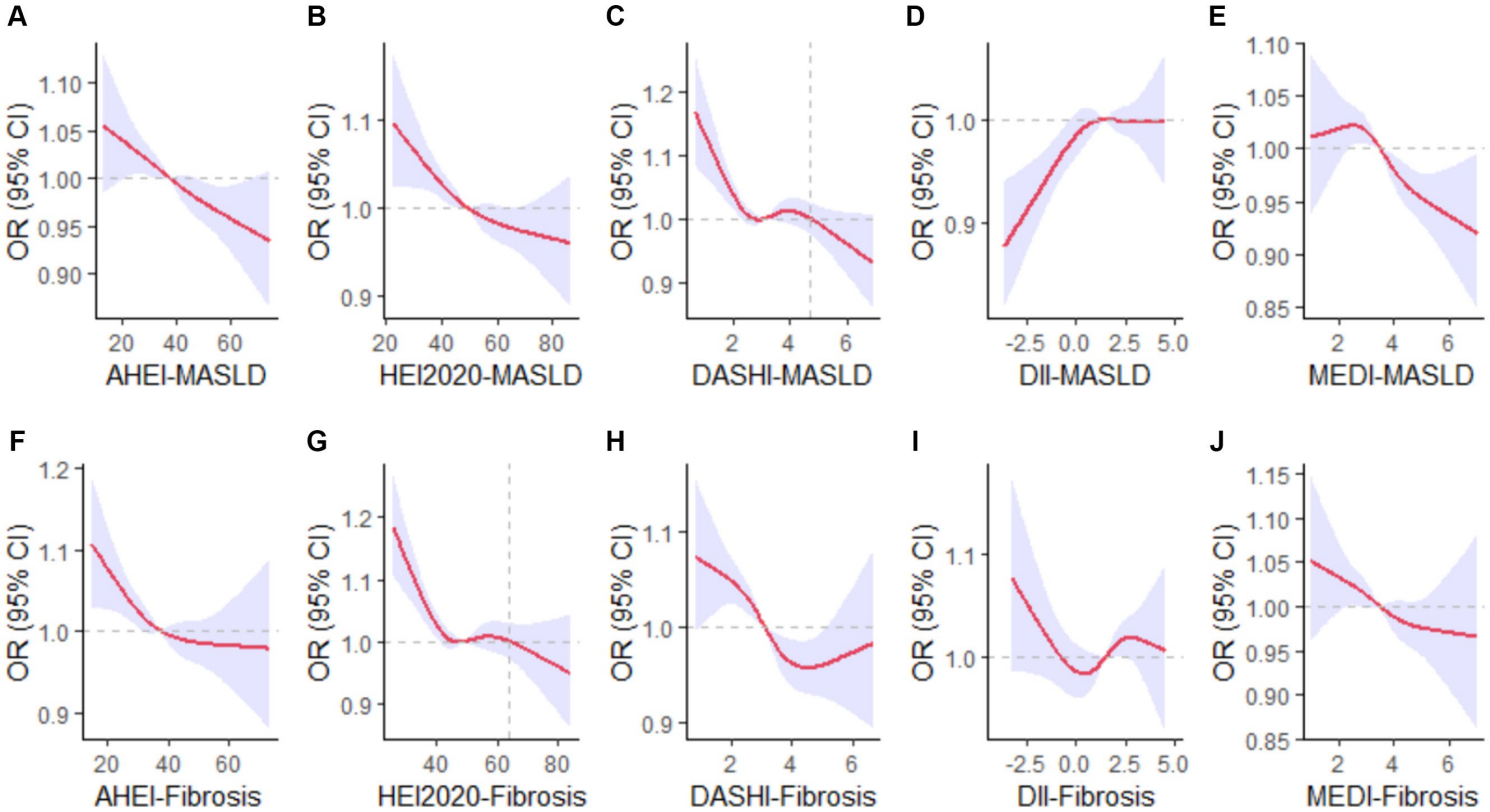
Restricted cubic spline curves for dietary indices and MASLD/MASLD-associated liver fibrosis. The red solid line indicates a smooth curve fit between the variables. The blue area indicates the 95% confidence interval of the fit. MASLD, metabolic dysfunction-associated steatotic liver disease; AHEI, Alternate Healthy Eating Index; HEI-2020, Healthy Eating Index-2020; DASHI, Dietary Approaches to Stop Hypertension index in serving sizes adapted from the DASH trial; DII, Dietary Inflammatory Index; MEDI, Mediterranean diet index in serving sizes from the PREDIMED trial. **(A–E)** Visualized relationships of AHEI, HEI-2020, DASHI, DII, MEDI with MASLD. **(F–J)** Visualized relationships of AHEI, HEI-2020, DASHI, DII, MEDI with MASLD-LF.

In the non-adjusted Model 1, all dietary indices were significantly correlated with MASLD. The AHEI, HEI-2020, DASHI, and MEDI were negatively correlated with the prevalence of MASLD, whereas DII was positively correlated. These correlations remained statistically significant after dividing all dietary indices into quartiles. The same conclusions still stood in Models 2 and 3 after the inclusion of different covariates, with different dietary indices affecting the risk of MASLD to varying degrees. Higher AHEI (continuous OR: 0.99; 95% CI: 0.98–0.99; p for trend: 0.002), HEI-2020 (continuous OR: 0.99; 95% CI: 0.98–0.99; p for trend: <0.001), DASHI (continuous OR: 0.89; 95% CI: 0.83–0.96; p for trend: 0.019), MEDI (continuous OR. 0.85; 95% CI: 0.75–0.97; p for trend: 0.012) reduced the risk of MASLD in Model 3, which was contrary to the case for DII (continuous OR: 1.09; 95% CI: 1.02–1.17; p for trend: 0.001)—as is further detailed in Table 2. Smooth curve-fitting further suggested no nonlinear association between AHEI (*p*-overall: 0.0021, *p*-nonlinear: 0.9682), HEI-2020 (*p*-overall: 0.0002, *p*-nonlinear: 0.3982), DII (*p*-overall: 0.0009, *p*-nonlinear: 0.0521), MEDI (*p*-overall: 0.0001, *p*-nonlinear: 0.3464) with MASLD; however, there was a temporary plateau in the RCS curve of DASHI versus MASLD, with a lower risk of MASLD at DASHI >4.70 compared to the reference point (*p*-overall: 0.0001, *p*-nonlinear: 0.0066; Figures 2A–E).

**Table 2:** Relations between dietary indices and MASLD.

	Model 1			Model 2			Model 3		
	OR	95% CI	*p*	OR	95% CI	*p*	OR	95% CI	*p*
AHEI									
Continuos	0.98	(0.97, 0.99)	**<0.001**	0.97	(0.96, 0.98)	**<0.001**	0.99	(0.98, 0.99)	**<0.001**
Quartile			<0.001*			<0.001*			0.002*
Q1 (8.28, 30.63)	Ref.			Ref.			Ref.		
Q2 (30.63, 38.70)	1.04	(0.79, 1.36)	0.796	0.92	(0.70, 1.21)	0.537	0.80	(0.58, 1.11)	0.141
Q3 (38.70, 46.79)	0.94	(0.75, 1.17)	0.544	0.75	(0.59, 0.96)	**0.023**	0.73	(0.53, 1.01)	0.055
Q4 (46.79, 87.29)	0.62	(0.50, 0.77)	**<0.001**	0.45	(0.36, 0.58)	**<0.001**	0.64	(0.46, 0.89)	**0.017**
HEI-2020									
Continuos	0.98	(0.98, 0.99)	**<0.001**	0.98	(0.97, 0.98)	**<0.001**	0.99	(0.98, 0.99)	**<0.001**
Quartile			<0.001*			<0.001*			<0.001*
Q1 (18.74, 40.94)	Ref.			Ref.			Ref.		
Q2 (40.94, 49.04)	0.90	(0.68, 1.18)	0.412	0.80	(0.60, 1.07)	0.125	0.72	(0.47, 1.11)	0.111
Q3 (49.04, 58.75)	0.88	(0.69, 1.11)	0.264	0.75	(0.58, 0.97)	**0.033**	0.73	(0.48, 1.10)	0.102
Q4 (58.75, 91.33)	0.62	(0.49, 0.79)	**<0.001**	0.48	(0.38, 0.60)	**<0.001**	0.61	(0.46, 0.80)	**0.006**
DASHI									
Continuos	0.84	(0.78, 0.92)	**<0.001**	0.81	(0.74, 0.89)	**<0.001**	0.89	(0.83, 0.96)	**<0.001**
Quartile			<0.001*			<0.001*			0.019*
Q1 (0.53, 2.47)	Ref.			Ref.			Ref.		
Q2 (2.47, 3.18)	0.70	(0.54, 0.90)	**0.009**	0.69	(0.52, 0.92)	**0.014**	0.80	(0.51, 1.24)	0.242
Q3 (3.18, 4.02)	0.71	(0.54, 0.93)	**0.014**	0.68	(0.51, 0.90)	**0.012**	0.81	(0.54, 1.22)	0.245
Q4 (4.02, 7.59)	0.60	(0.47, 0.76)	**<0.001**	0.53	(0.41, 0.69)	**<0.001**	0.74	(0.58, 0.95)	**0.026**
DII									
Continuos	1.06	(1.02, 1.11)	**0.006**	1.13	(1.07, 1.18)	**<0.001**	1.09	(1.02, 1.17)	**0.003**
Quartile			<0.001*			<0.001*			0.001*
Q1 (−4.82, 0.09)	Ref.			Ref.			Ref.		
Q2 (0.09, 1.35)	1.37	(1.14, 1.65)	**0.002**	1.55	(1.25, 1.92)	**<0.001**	1.65	(1.13, 2.42)	**0.020**
Q3 (1.35, 2.61)	1.51	(1.19, 1.93)	**0.002**	1.80	(1.40, 2.31)	**<0.001**	1.57	(1.10, 2.24)	**0.022**
Q4 (2.61, 4.80)	1.23	(0.98, 1.54)	0.074	1.62	(1.28, 2.05)	**<0.001**	1.47	(1.01, 2.13)	**0.046**
MEDI									
Continuos	0.84	(0.76, 0.92)	**<0.001**	0.81	(0.74, 0.89)	**<0.001**	0.85	(0.75, 0.97)	**0.004**
Quartile			<0.001*			<0.001*			0.012*
Q1 (0.5, 3.0)	Ref.			Ref.			Ref.		
Q2 (3.0, 3.5)	0.81	(0.62, 1.05)	0.101	0.80	(0.61, 1.06)	0.11	0.83	(0.52, 1.30)	0.331
Q3 (3.5, 4.5)	0.80	(0.65, 0.99)	**0.037**	0.75	(0.60, 0.93)	**0.013**	0.79	(0.59, 1.08)	0.11
Q4 (4.5, 8.0)	0.55	(0.39, 0.78)	**0.002**	0.51	(0.35, 0.72)	**0.001**	0.56	(0.34, 0.94)	**0.036**

OR, Odds ratio; CI, Confidence interval; AHEI, Alternate Healthy Eating Index; HEI-2020, Healthy Eating Index-2020; DASHI, Dietary Approaches to Stop Hypertension index in serving sizes adapted from the DASH trial; DII, Dietary Inflammatory Index; MED, Mediterranean diet index in serving sizes from the PREDIMED trial. Model 1: No covariates were adjusted. Model 2: Adjusted for age, sex, race, and education. Model 3: Adjusted for age, sex, race, education, BMI grade, smoking status, hypertension, diabetes, TG, TC, and HDL-C. *p for trends.Bold values indicate statistical significance.

### 3.3 Association of dietary indices with MASLD-LF

The effects of the five dietary indices on MASLD-LF in the population with MASLD were analyzed using the three models under the same covariate conditions. Significant negative associations between AHEI, HEI-2020, DASHI, and MEDI with MASLD-LF were found in Models 1 and 2, with interquartile trends (*p* < 0.05) only for HEI-2020 and DASHI. In Model 3, as continuous variables, higher HEI-2020 (continuous OR: 0.98; 95% CI: 0.95–1.00) and DASHI (continuous OR: 0.79; 95% CI: 0.64–0.97) were still associated with a lower risk of fibrosis. Compared to the lowest quartile, the risk of MASLD-LF was reduced by 60% (OR: 0.40; 95% CI: 0.21–0.76; *p* for trend: 0.003) in the highest DASHI quartile (Table 3). The RCS results suggested a nonlinear association between HEI-2020 and MASLD-LF (*p*-overall: <0.0001, *p*-nonlinear: 0.0022) and a reduced risk of fibrosis when HEI-2020 was >64.07 vs. the reference point. No nonlinear associations were found in terms of AHEI (*p*-overall: 0.0040, *p*-nonlinear: 0.2330) and DASHI (*p*-overall: <0.0001, *p*-nonlinear: 0.1181), with MASLD-LF. No correlation with fibrosis was found for DII (*p*-overall: 0.1962, *p*-nonlinear: 0.0998) and MEDI (*p*-overall: 0.0671, *p*-nonlinear: 0.8410)—as is further detailed in Figures 2F–J.

**Table 3:** Relations between dietary indices and MASLD-associated liver fibrosis.

	Model 1			Model 2			Model 3		
	OR	95% CI	*p*	OR	95% CI	*p*	OR	95% CI	*p*
AHEI									
Continuos	0.98	(0.96, 1.00)	**0.008**	0.97	(0.95, 1.00)	**0.008**	0.98	(0.95, 1.01)	0.071
Quartile			0.094*			0.089*			0.234*
Q1 (9.39, 30.07)	Ref.			Ref.			Ref.		
Q2 (30.07, 37.70)	0.84	(0.54, 1.32)	0.427	0.81	(0.50, 1.30)	0.356	0.74	(0.36, 1.51)	0.329
Q3 (37.70, 45.13)	0.59	(0.31, 1.14)	0.112	0.55	(0.27, 1.12)	0.095	0.53	(0.23, 1.21)	0.104
Q4 (45.13, 81.34)	0.55	(0.32, 0.94)	**0.032**	0.49	(0.26, 0.94)	**0.033**	0.59	(0.25, 1.42)	0.184
HEI-2020									
Continuos	0.98	(0.96, 0.99)	**0.002**	0.97	(0.96, 0.99)	**0.003**	0.98	(0.95, 1.00)	**0.029**
Quartile			0.021*			0.029*			0.109*
Q1 (20.35, 40.21)	Ref.			Ref.			Ref.		
Q2 (40.21, 47.81)	0.57	(0.30, 1.07)	0.077	0.55	(0.28, 1.08)	0.080	0.52	(0.24, 1.14)	0.086
Q3 (47.81, 57.33)	0.62	(0.34, 1.14)	0.119	0.61	(0.30, 1.22)	0.147	0.63	(0.27, 1.48)	0.223
Q4 (57.33, 91.33)	0.48	(0.30, 0.79)	**0.006**	0.46	(0.26, 0.82)	**0.012**	0.51	(0.23, 1.12)	0.079
DASHI									
Continuos	0.75	(0.64, 0.88)	**<0.001**	0.75	(0.64, 0.90)	**<0.001**	0.79	(0.64, 0.97)	**0.006**
Quartile			<0.001*			<0.001*			0.003*
Q1 (0.53, 2.33)	Ref.			Ref.			Ref.		
Q2 (2.33, 3.08)	0.70	(0.46, 1.08)	0.103	0.70	(0.45, 1.10)	0.115	0.71	(0.43, 1.18)	0.142
Q3 (3.08, 3.90)	0.59	(0.37, 0.95)	**0.033**	0.59	(0.36, 0.96)	**0.036**	0.67	(0.35, 1.28)	0.172
Q4 (3.90, 7.53)	0.37	(0.24, 0.58)	**<0.001**	0.37	(0.23, 0.60)	**<0.001**	0.40	(0.21, 0.76)	**0.015**
DII									
Continuos	1.03	(0.91, 1.17)	0.580	1.05	(0.91, 1.19)	0.484	1.00	(0.84, 1.20)	0.947
Quartile			0.526*			0.460*			0.628*
Q1 (−4.09, 0.19)	Ref.			Ref.			Ref.		
Q2 (0.19, 1.44)	0.98	(0.63, 1.54)	0.944	1.01	(0.63, 1.63)	0.962	0.99	(0.57, 1.74)	0.976
Q3 (1.44, 2.59)	1.00	(0.60, 1.68)	0.992	1.04	(0.61, 1.79)	0.877	0.91	(0.45, 1.85)	0.759
Q4 (2.59, 4.65)	1.39	(0.81, 2.39)	0.224	1.47	(0.81, 2.66)	0.185	1.29	(0.60, 2.77)	0.438
MEDI									
Continuos	0.84	(0.73, 0.98)	**0.017**	0.85	(0.73, 0.99)	**0.028**	0.85	(0.71, 1.03)	0.042
Quartile			0.231*			0.232*			0.328*
Q1 (0.5, 3.0)	Ref.			Ref.			Ref.		
Q2 (3.0, 3.5)	0.90	(0.50, 1.63)	0.727	0.91	(0.49, 1.69)	0.757	0.91	(0.47, 1.76)	0.721
Q3 (3.5, 4.0)	0.81	(0.50, 1.30)	0.363	0.82	(0.48, 1.37)	0.416	0.81	(0.39, 1.67)	0.484
Q4 (4.0, 8.0)	0.63	(0.39, 1.00)	0.051	0.63	(0.39, 1.02)	0.061	0.66	(0.37, 1.18)	0.123

OR, Odds ratio; CI, Confidence interval; AHEI, Alternate Healthy Eating Index; HEI-2020, Healthy Eating Index-2020; DASHI, Dietary Approaches to Stop Hypertension index in serving sizes adapted from the DASH trial; DII, Dietary Inflammatory Index; MED, Mediterranean diet index in serving sizes from the PREDIMED trial. Model 1, No covariates were adjusted. Model 2, Adjusted for age, sex, race, and education. Model 3, Adjusted for age, sex, race, education, BMI grade, smoking status, hypertension, diabetes, TG, TC, and HDL-C. *p for trends.Bold values indicate statistical significance.

### 3.4 Subgroup analysis

Further, subgroup analyses were performed to assess the robustness of the results. No interaction was found across the subgroups for the five dietary indices with MASLD (Supplementary Figures S1–S5). The non-Hispanic White and more highly educated subgroups did show robust correlations with these parameters (*p*-interaction: >0.05). By contrast, the effects of HEI-2020 and DII on MASLD-LF were found to be significantly stratified according to educational level, hypertension, and ethnicity (*p*-interaction: <0.05; Supplementary Figures S6–S10). No interactions were found for any of the other variables.

## 4 Discussion

Specific dietary components or patterns have been mentioned as being associated with NAFLD in several previous studies, and dietary patterns such as low-calorie, Mediterranean, and high-quality diets have been suggested to be associated with a reduced risk of NAFLD (10, 25, 26). Since the Delphi Consensus Statement proposed MASLD as a new definition of steatotic liver disease, to replace NAFLD, it has not been confirmed whether the conclusions of previous studies on NAFLD are equally applicable to MASLD.

At baseline, nearly half of the population was found to have MASLD, diet quality assessed by the dietary indices was generally low, and Mexican Americans appeared to be more susceptible to MASLD, consistent with previous studies (11, 17, 27). Epidemiologic studies have shown that Mexican Americans have higher rates of obesity and prevalence of metabolic syndrome than other ethnicities (28, 29). In addition, some studies have reported that variants in the susceptibility gene for MASLD, PNPLA3, are more common among Mexican populations (30). As such, the prevalence of MASLD in specific populations appears to result from a complex interaction of behavioral and genetic factors that are not fully understood.

In this study, the associations of the five dietary pattern indices with MASLD and MASLD-LF were evaluated in a nationally-representative sample of adults living in the U.S. We found that AHEI, HEI-2020, DASHI, and MEDI were negatively associated with the risk of developing MASLD, whereas DII was positively associated. Anti-inflammatory diets reduced the risk of MASLD; however, a higher positive DII did not further increase MASLD risk. Among these, the highest quartile of MEDI scores was associated with the greatest reduction in MASLD risk compared to the lowest quartile, up to 44% (Q1 vs. Q4 OR: 0.56). These results are similar to previously reported associations between different definitions of fatty liver and dietary indices (10, 17, 31, 32). However, in our analyses related to fibrosis, only DASHI was found to be significantly associated with MASLD-LF on both continuous and quartile scales.

Smoothed curve-fitting visually demonstrated the relationship between dietary indices and MASLD traits. Our subgroup analyses identified a small number of variables that interacted with MASLD-LF—including the educational level and hypertension subgroups in the HEI-2020—suggesting that caution should be exercised when interpreting the association between HEI-2020 and MASLD-LF. We found robust associations in multiple subgroup analyses between the Dietary Index-MASLD for non-Hispanic White and highly educated populations. On the one hand, we considered that this result may have been related to other factors such as energy intake and socioeconomic status. For example, the AHS-2 cohort study found that healthy dietary patterns were less effective for preventing cardiovascular risk factors in Black populations compared to non-Hispanic White ones (33), while another cohort study reported that the Black population had a significantly higher energy intake than the White population across all dietary groups (34). These findings suggest that the effect of dietary indices vary across different ethnicities, with differences in energy intake serving as a possible explanation. Additionally, people with higher educational levels tend to have higher socioeconomic statuses, and therefore be more likely to have access to healthier diets and better health care. On the other hand, the large sample sizes of these two groups may have increased their statistical power, making the observed associations more significant and stable. This tendency was also observed in another study (35).

When comparing the components of the different dietary indices, it can be seen that AHEI, HEI-2020, DASHI, and MEDI all encourage eating more vegetables, fruits, whole grains, nuts, proteins, and mono-and polyunsaturated fatty acids (MUFA/PUFA) while restricting the consumption of red meat (MEDI), processed meat (AHEI, MEDI), SSB (AHEI, MEDI), sodium (AHEI, HEI-2020, DASHI), and saturated fatty acids (SFA) (HEI-2020, DASHI). Previous studies have reported that the excessive intake of saturated fat, SSB, red and processed meat, and sodium promotes hepatic fat accumulation (6, 36). SSBs are rich in fructose and are metabolized by the liver. Compared to glucose, fructose is more likely to be a substrate for *de novo* lipogenesis, as it is metabolized independently of insulin and cellular energy status. The excessive intake of fructose therefore induces hepatic fat accumulation and further triggers hepatic and systemic insulin resistance (IR) via endoplasmic reticulum stress and other pathways (37, 38). High-sodium diets can also induce IR by increasing plasma free fatty acid levels and white adipose tissue mass. White adipose tissue secretes leptin and inflammatory factors that exacerbate IR and hepatic inflammation (36). Several cross-sectional and longitudinal studies have found a positive correlation between SFA and hepatic fat, and a negative correlation between PUFA and hepatic fat (39, 40). Compared to PUFA, SFA leads to hepatic steatosis by decreasing whole-body oxidation and insulin-mediated inhibition of lipolysis upon entry into the human body (41), thus increasing hepatic and other visceral fat deposition, as well as weight gain (42). Therefore, dietary patterns that restrict SFA intake and promote the intake of PUFA-rich foods, such as nuts and olive oil, are associated with a reduced risk of MASLD.

Our study found a robust negative association between DASHI score and MASLD-LF risk. The DASHI is the only one of the five scores that were included in this study. It is calculated based on nutrients and emphasizes the intake of minerals and dietary fiber, in addition to the restriction of sodium and SFA intake (which are all mentioned in the other scores as well). Only a few cross-sectional studies have reported that higher intakes of soluble dietary fiber and magnesium are associated with a reduced risk of advanced fibrosis (43, 44). The mechanism by which DASH significantly reduces the risk of MASLD-LF compared to other dietary patterns remains to be elucidated in further studies. As for the other scores were not associated with MASLD-fibrosis for some reason. We also do not except the possibility that dietary factors may influence liver fibrosis in specific situations so that we were unable to find such a correlation in the overall population. For example, patients with MASLD who possess a susceptibility mutation in of PNPLA3 are also more sensitive to liver steatosis mediated by dietary factors (45), and further studies are needed to elaborate on the association between diet and liver fibrosis in this population.

The major strengths of this study include its being the first to analyze the associations of MASLD and MASLD-LF with five different dietary indices using a nationally representative sample from the most recent NHANES cycle, as well as applying the latest diagnostic and dietary index criteria to this analysis. Nevertheless, several key limitations should be noted as well. First, its cross-sectional design inherently provided only a low level of evidence to determine whether the associations between dietary indices and MASLD traits were causally related. Further studies are therefore warranted to validate these associations. Second, all of the dietary indices analyzed were calculated based on information from questionnaires, which may have introduced recall bias and therefore may not represent long-term dietary habits. Despite the large overall sample size of the study, certain subgroups (e.g., certain ethnic minorities and populations with lower educational levels) may have been under-represented, thus limiting accurate analyses in these subgroups. Finally, owing to the lack of diagnostic codes in our dataset, it was not possible to definitively exclude the potential presence of other comorbid diseases in the study population that may have also contributed to liver steatosis or fibrosis—such as autoimmune liver disease, Wilson’s disease, and hypobetalipoproteinemia. Although the determination of liver steatosis or fibrosis based on VCTE carries the advantages of being non-invasive and safe, it cannot replace liver biopsy because its accuracy is affected by obesity, ascites, and operator techniques. These factors may have introduced some degree of bias to the study. Future studies should consider these limitations and perform in-depth explorations of the mechanisms behind different dietary effects on MASLD traits.

## 5 Conclusion

The AHEI, HEI-2020, DASHI, DII, and MEDI nutritional indices were all found to be associated with the risk of developing MASLD in the United States population, whereas the DASHI was associated with the risk of MASLD-LF. Based on the results of this study, specific dietary habits may reduce the risk of MASLD and MASLD-LF. However, large-sample randomized controlled trials are warranted to validate these findings.

## Data Availability

Publicly available datasets were analyzed in this study. This data can be found at: https://wwwn.cdc.gov/nchs/nhanes/Default.aspx.
