# Supplementary figures and images for: Associations of five dietary indices with metabolic dysfunction-associated steatotic liver disease and liver fibrosis among the United States population
Source: Front Nutr. 2024 Aug 16;11:1446694. doi: 10.3389/fnut.2024.1446694 (PMC11363712; doi:10.3389/fnut.2024.1446694)

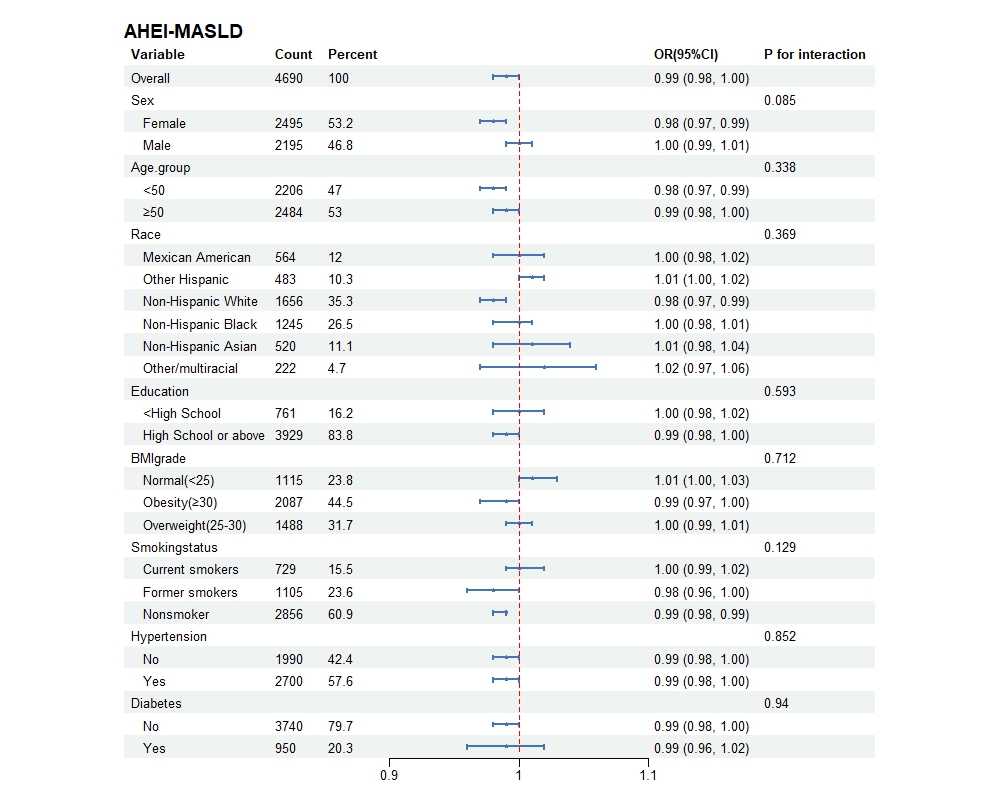

Supplement: Supplementary file 2 [file Image_1.JPEG]

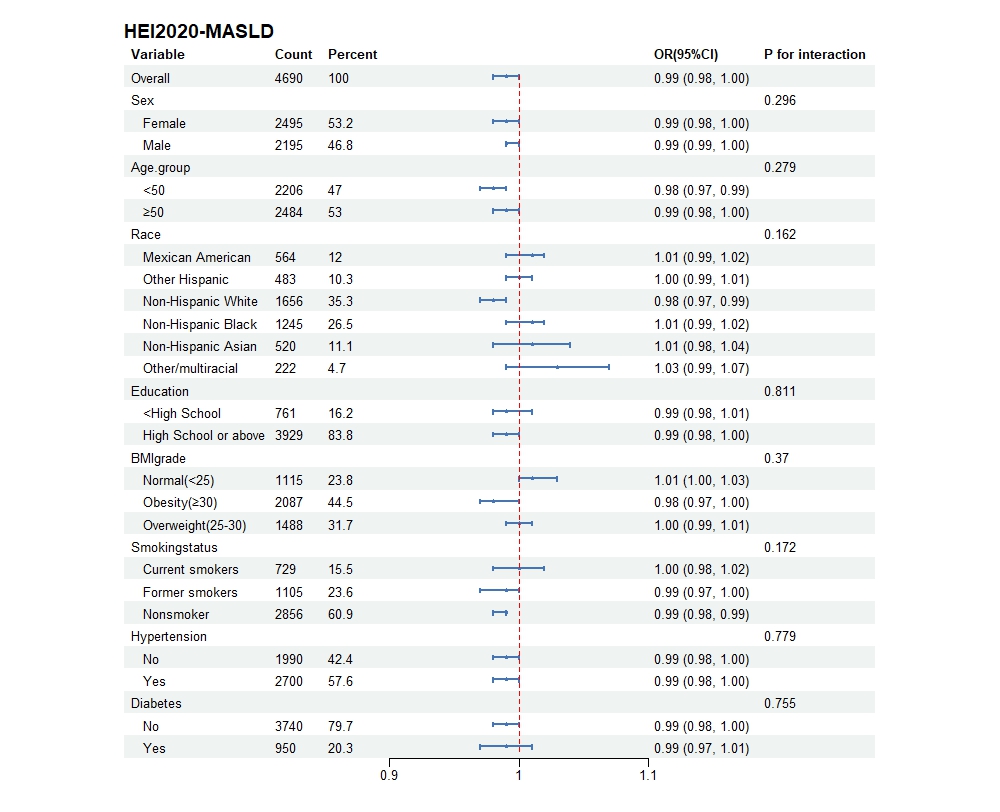

Supplement: Supplementary file 3 [file Image_2.JPEG]

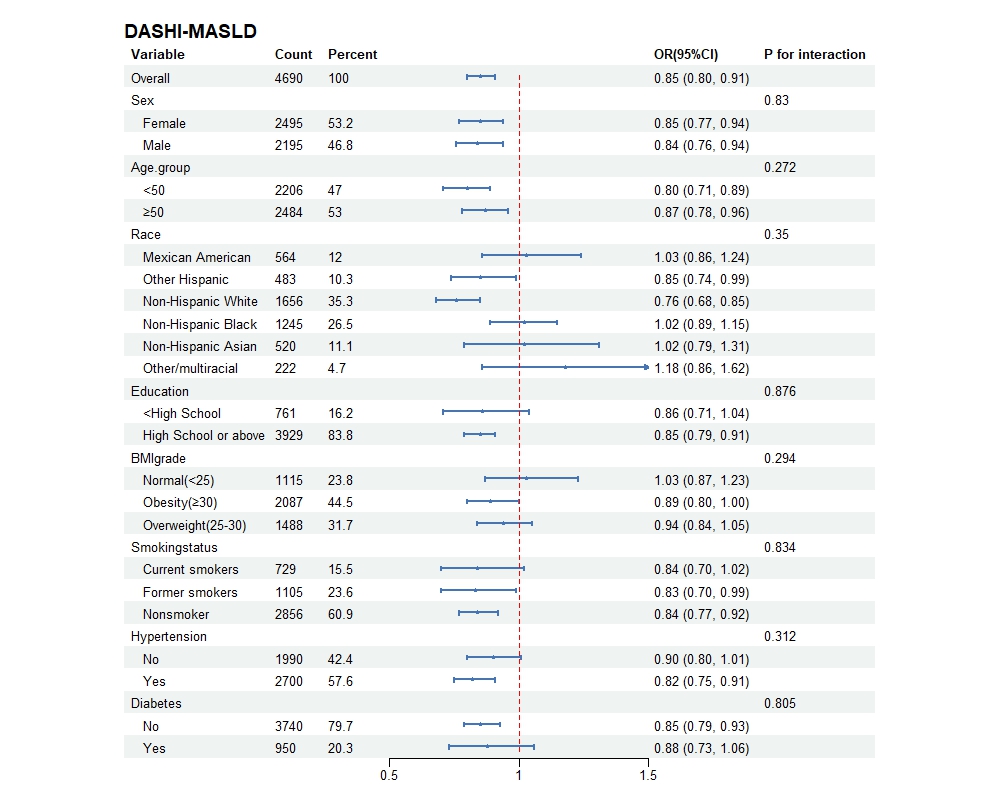

Supplement: Supplementary file 4 [file Image_3.JPEG]

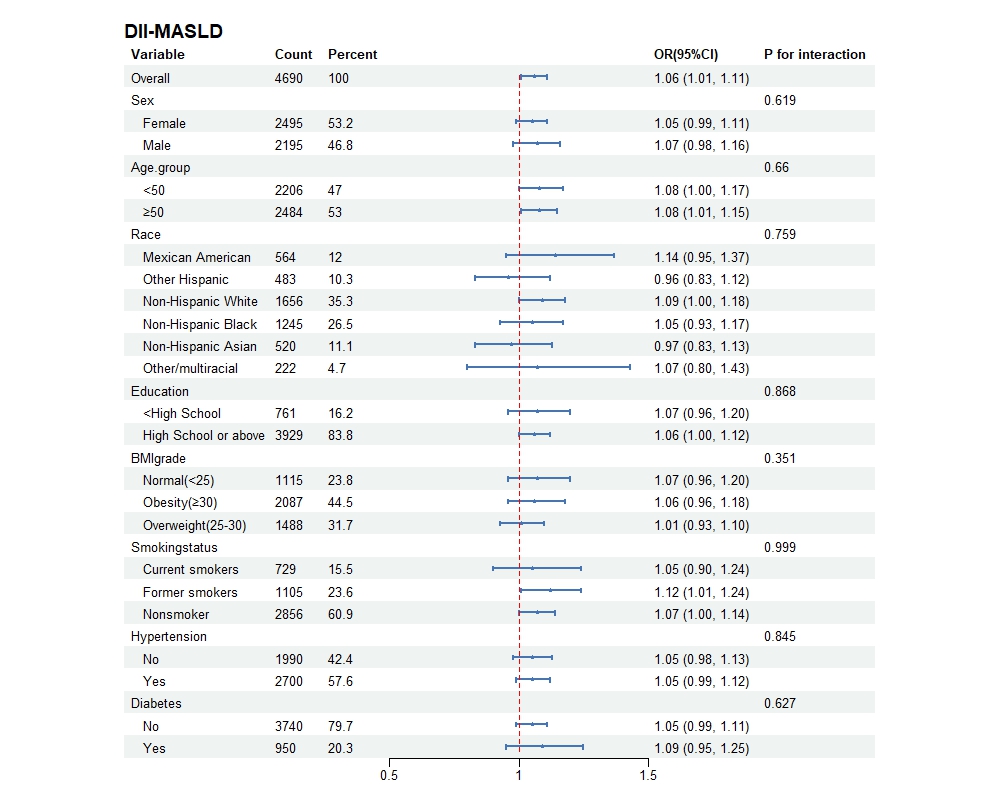

Supplement: Supplementary file 5 [file Image_4.JPEG]

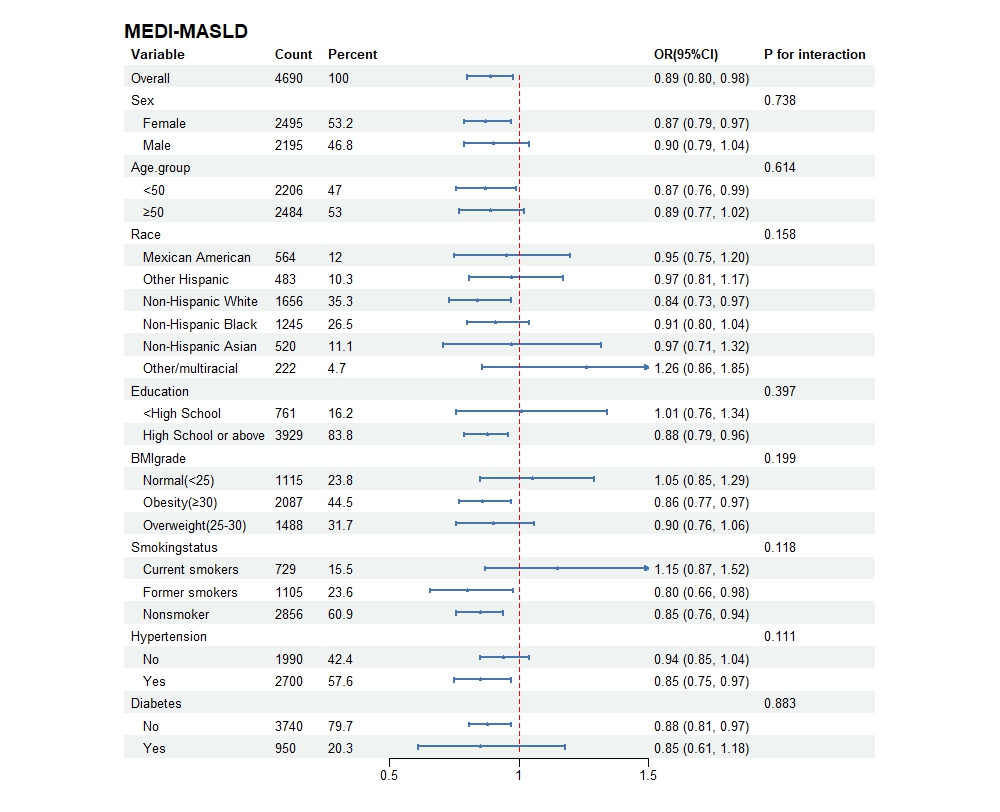

Supplement: Supplementary file 6 [file Image_5.JPEG]

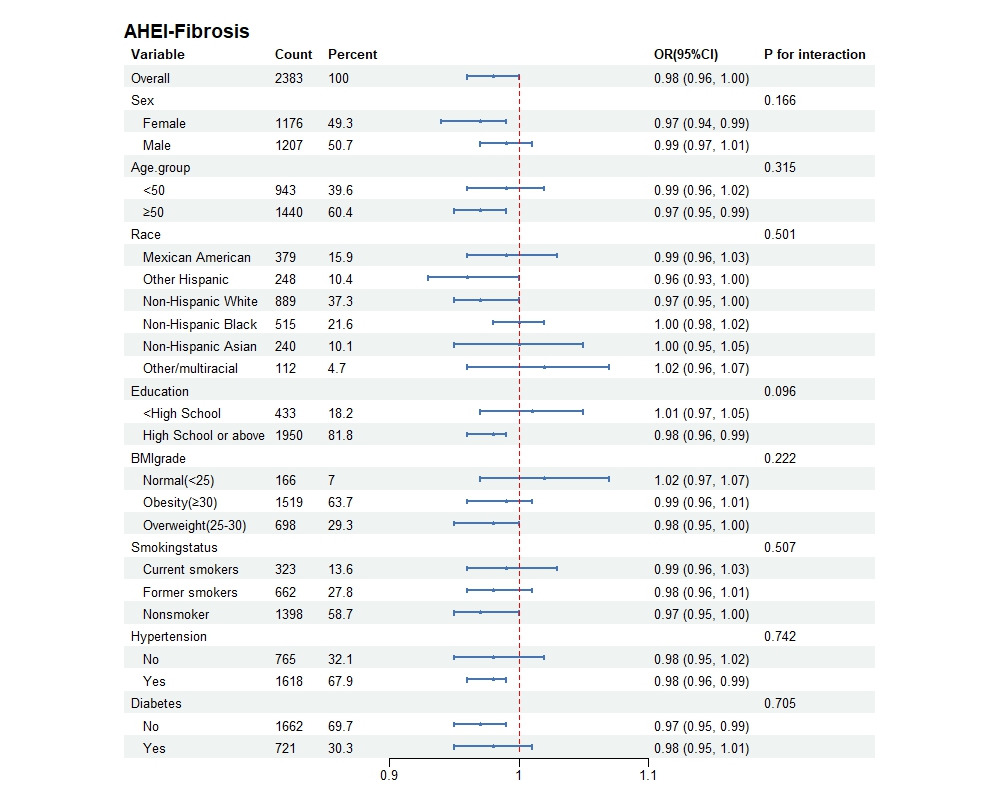

Supplement: Supplementary file 7 [file Image_6.JPEG]

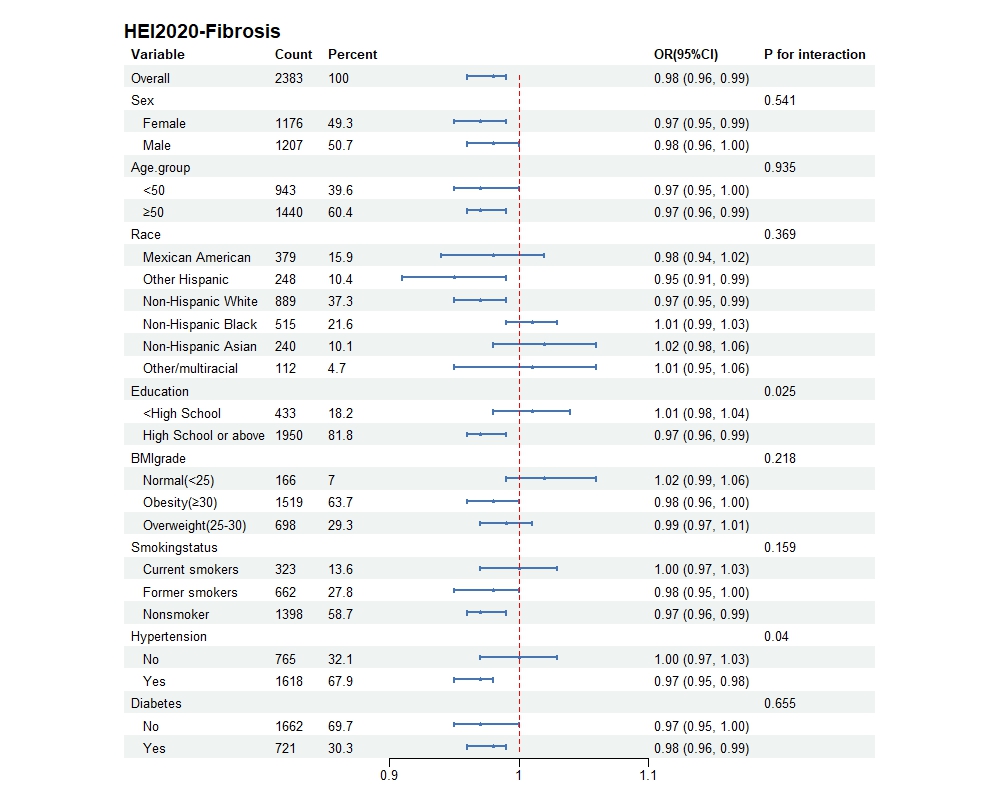

Supplement: Supplementary file 8 [file Image_7.JPEG]

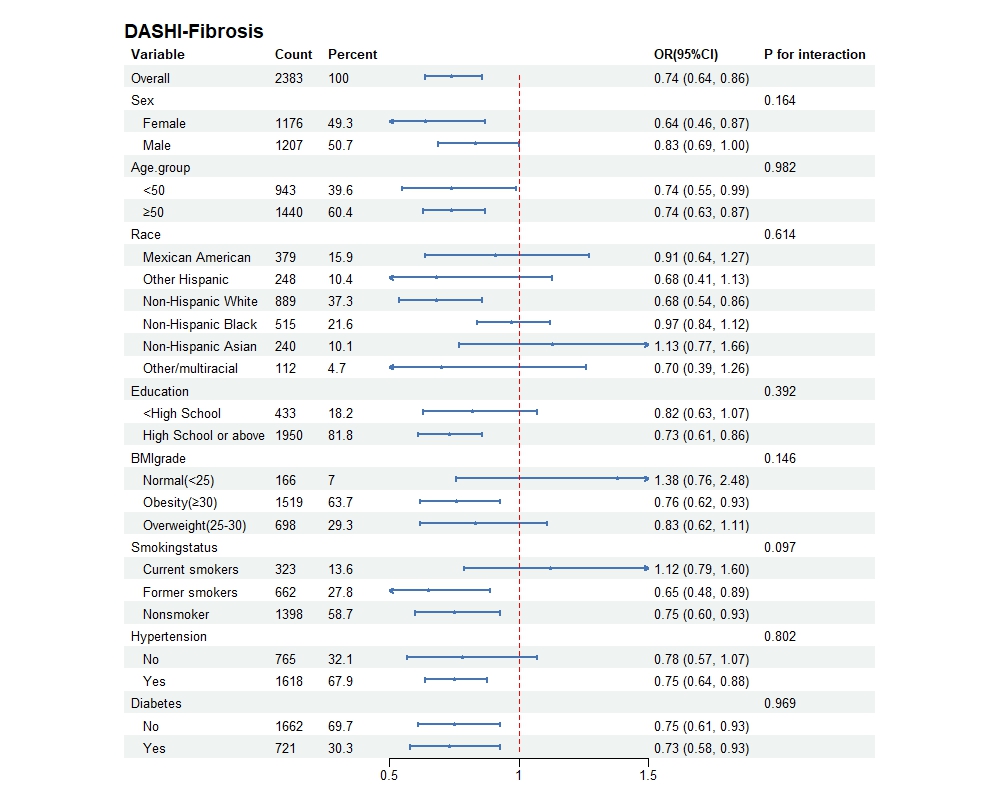

Supplement: Supplementary file 9 [file Image_8.JPEG]

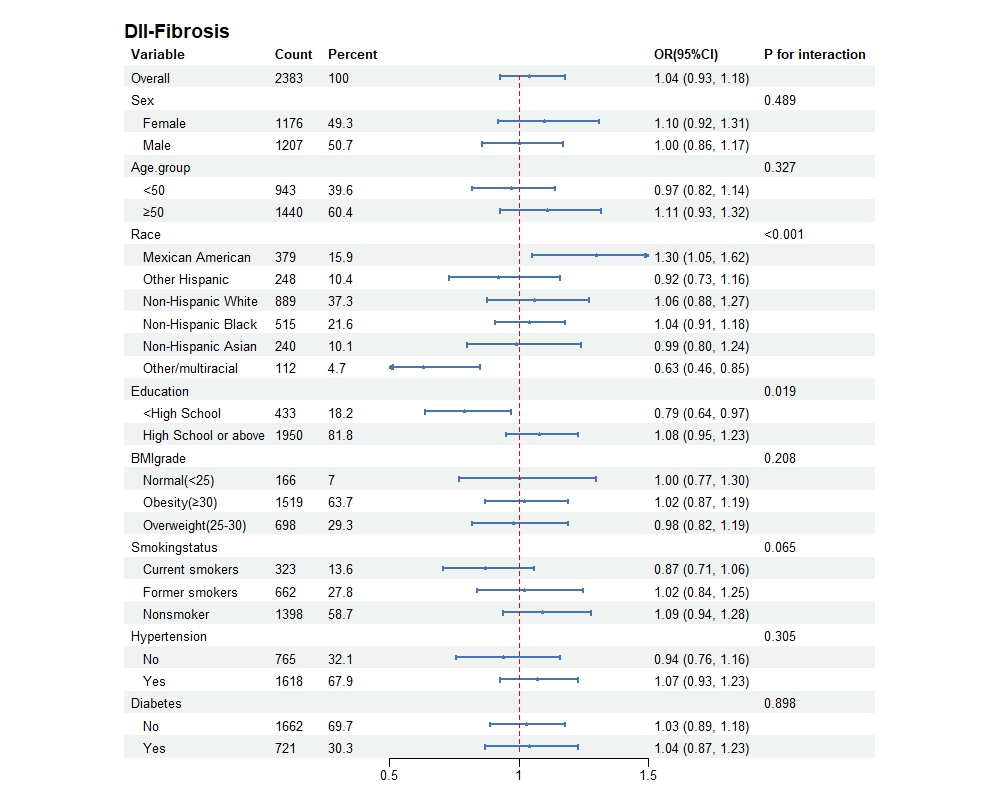

Supplement: Supplementary file 10 [file Image_9.JPEG]

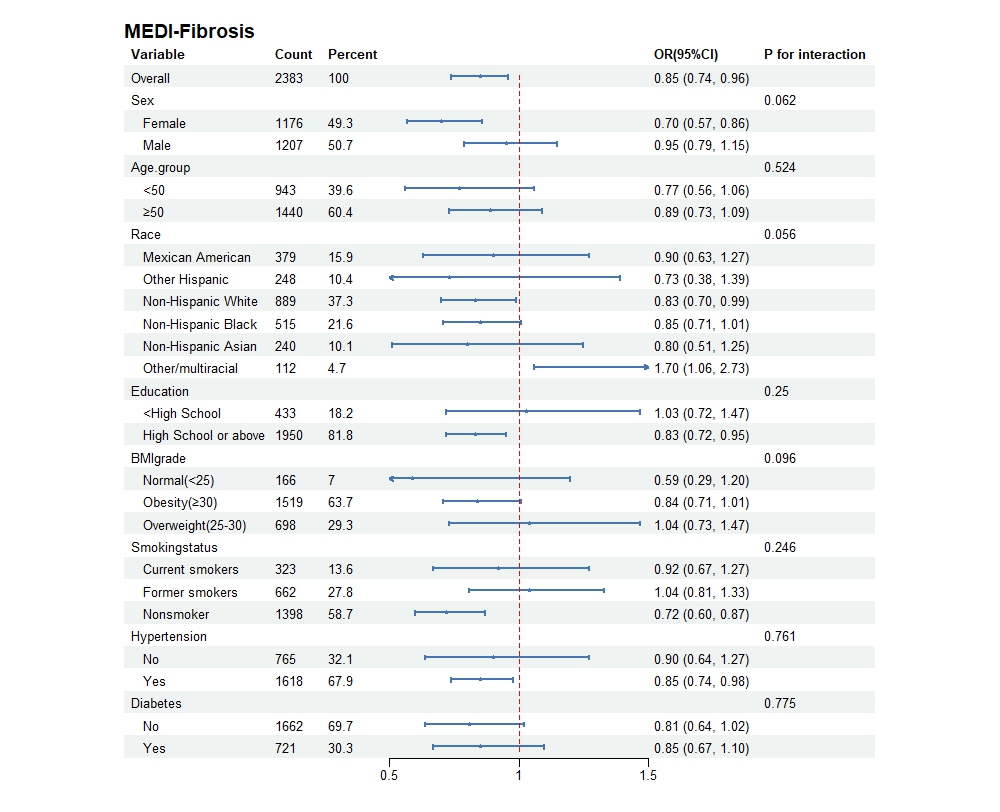

Supplement: Supplementary file 11 [file Image_10.JPEG]
